# Supplementary material for: Causal Relationship between Aging and Anorexia Nervosa: A White-Matter-Microstructure-Mediated Mendelian Randomization Analysis
Source: Biomedicines. 2024 Aug 16;12(8):1874. doi: 10.3390/biomedicines12081874 (PMC11351342; doi:10.3390/biomedicines12081874)
Supplement: Supplementary file 1 [file biomedicines-12-01874-s001.zip › Supplementary Figure/Figure S4/Singe SNP plot of MR analysis for TL to ubm-b-1879.pdf]

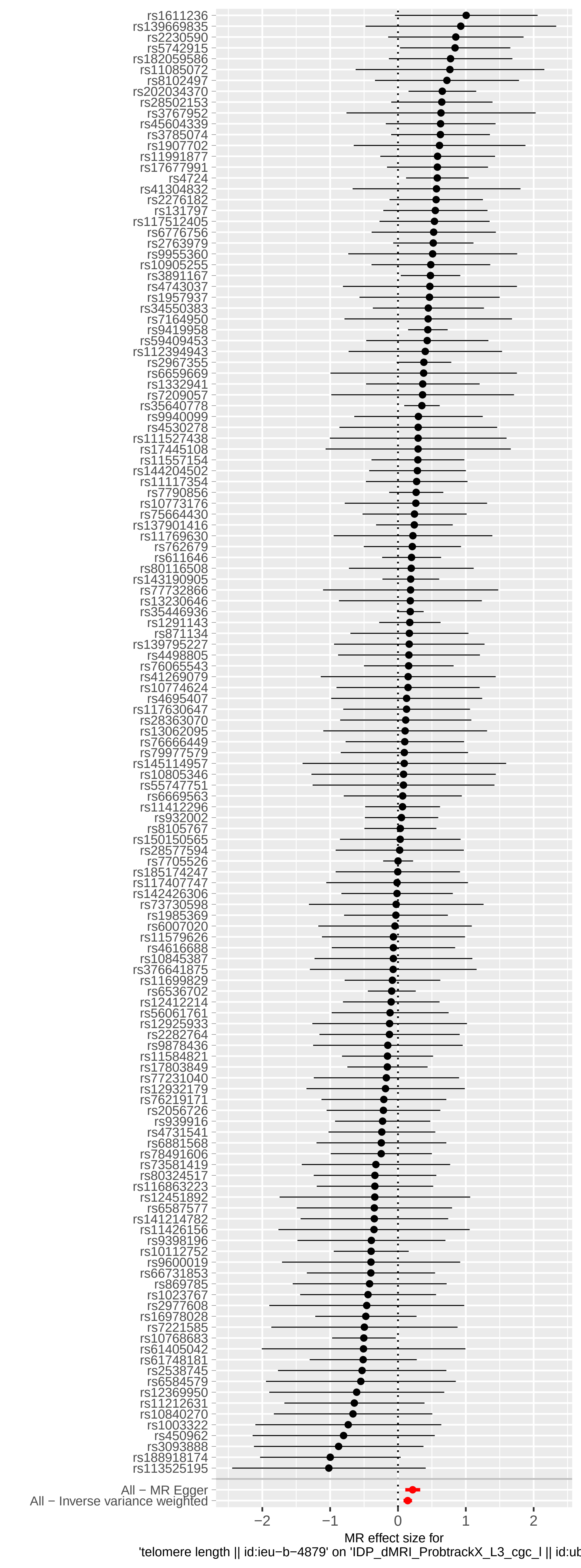
rs1611236
rs139669835
rs2230590
rs5742915
rs182059586
rs11085072
rs8102497
rs202034370
rs28502153
rs3767952
rs45604339
rs3785074
rs1907702
rs11991877
rs17677991
rs4724
rs41304832
rs2276182
rs131797
rs117512405
rs6776756
rs2763979
rs9955360
rs10905255
rs3891167
rs4743037
rs1957937
rs34550383
rs7164950
rs9419958
rs59409453
rs112394943
rs2967355
rs6659669
rs1332941
rs7209057
rs35640778
rs9940099
rs4530278
rs111527438
rs17445108
rs11557154
rs144204502
rs11117354
rs7790856
rs10773176
rs75664430
rs137901416
rs11769630
rs762679
rs611646
rs80116508
rs143190905
rs77732866
rs13230646
rs35446936
rs1291143
rs871134
rs139795227
rs4498805
rs76065543
rs41269079
rs10774624
rs4695407
rs117630647
rs28363070
rs13062095
rs76666449
rs79977579
rs145114957
rs10805346
rs55747751
rs6669563
rs11412296
rs932002
rs8105767
rs150150565
rs28577594
rs7705526
rs185174247
rs117407747
rs142426306
rs73730598
rs1985369
rs6007020
rs11579626
rs4616688
rs10845387
rs376641875
rs11699829
rs6536702
rs12412214
rs56061761
rs12925933
rs2282764
rs9878436
rs11584821
rs17803849
rs77231040
rs12932179
rs76219171
rs2056726
rs939916
rs4731541
rs6881568
rs78491606
rs73581419
rs80324517
rs116863223
rs12451892
rs6587577
rs141214782
rs11426156
rs9398196
rs10112752
rs9600019
rs66731853
rs869785
rs1023767
rs2977608
rs16978028
rs7221585
rs10768683
rs61405042
rs61748181
rs2538745
rs6584579
rs12369950
rs11212631
rs10840270
rs1003322
rs450962
rs3093888
rs188918174
rs113525195
All − MR Egger
All − Inverse variance weighted
−2
−1
0
1
2
MR effect size for
'telomere length || id:ieu−b−4879' on 'IDP_dMRI_ProbtrackX_L3_cgc_l || id:ub
